# Water-Soluble Ruthenium(III)-Dimethyl Sulfoxide Complexes: Chemical Behaviour and Pharmaceutical Properties

**DOI:** 10.1155/MBD.1994.41

**Published:** 1994

**Authors:** G. Mestroni, E. Alessio, G. Sava, S. Pacor, M. Coluccia, A. Boccarelli

**Affiliations:** 1 Department of Chemical Sciences School of Pharmacy University of Trieste Trieste I-34127 Italy; 2 Institute of Pharmacology School of Pharmacy University of Trieste Trieste I-34127 Italy; 3 Department of Biomedical Sciences and Human Oncology University of Bari Bari I-70124 Italy

## Abstract

In this paper we report a review of the results obtained in the last few years by our group in the development of ruthenium(III) complexes characterized by the presence of sulfoxide ligands and endowed with antitumor properties. In particular, we will focus on ruthenates of general formula Na[*trans*-RuCl_4_(R_1_R_2_SO)(L)], where R_1_R_2_SO = dimethylsulfoxide (DMSO) or tetramethylenesulfoxide (TMSO) and L = nitrogen donor ligand. The chemical behavior of these complexes has been studied by means of spectroscopic techniques both in slightly acidic distilled water and in phosphate buffered solution at physiological pH. The influence of biological reductants on the chemical behavior is also described. The antitumor properties have been investigated on a number of experimental tumors. Out of the effects observed, notheworthy appears the capability of the tested ruthenates to control the metastatic dissemination of solid metastasizing tumors. The analysis of the antimetastatic action, made in particular on the MCa mammary carcinoma of CBA mouse, has demonstrated a therapeutic value for these complexes which are able to significantly prolong the survival time of the treated animals. The antimetastatic effect is not attributable to a specific cytotoxicity for metastatic tumor cells although *in vitro* experiments on pBR322 double stranded DNA has shown that the test ruthenates bind to the macromolecule, causing breaks corresponding to almost all bases, except than thymine, and are able to cause interstrand bonds, depending on the nature of the complex being tested, some of which results active as cisplatin itself.


### WATER-SOLUBLE RUTHENIUM(III)-DIMETHYL SULFOXIDE COMPLEXES: CHEMICAL BEHAVIOUR AND PHARMACEUTICAL PROPERTIES

G. Mestroni[1], E. Alessio[1], G, Sava[2], S. Pacor[2], M. Coluccia[3] and A. Boccarelli[3]

[1] Department of Chemical Sciences, and [2] Institute of Pharmacology, School of Pharmacy, University of Trieste, I-34127 Trieste, Italy
[3] Department of Biomedical Sciences and Human Oncology, University of Bari, I-70124 Bari, Italy

### Abstract

In this paper we report a review of the results obtained in the last few years by our group in the development of ruthenium(III) complexes characterized by the presence of sulfoxide ligands and endowed with antitumor properties. In particular, we will focus on ruthenates of general formula Na[*trans*-$RuCl_4(R_1R_2SO)(L)$], where $R_1R_2SO$ = dimethylsulfoxide (DMSO) or tetramethylenesulfoxide (TMSO) and L = nitrogen donor ligand. The chemical behavior of these complexes has been studied by means of spectroscopic techniques both in slightly acidic distilled water and in phosphate buffered solution at physiological pH. The influence of biological reductants on the chemical behavior is also described. The antitumor properties have been investigated on a number of experimental tumors. Out of the effects observed, notheworthy appears the capability of the tested ruthenates to control the metastatic dissemination of solid metastasizing tumors. The analysis of the antimetastatic action, made in particular on the MCa mammary carcinoma of CBA mouse, has demonstrated a therapeutic value for these complexes which are able to significantly prolong the survival time of the treated animals. The antimetastatic effect is not attributable to a specific cytotoxicity for metastatic tumor cells although *in vitro* experiments on pBR322 double stranded DNA has shown that the test ruthenates bind to the macromolecule, causing breaks corresponding to almost all bases, except than thymine, and are able to cause interstrand bonds, depending on the nature of the complex being tested, some of which results active as cisplatin itself.

### Introduction

Despite the leading role played by *cis*-dichlorodiammineplatinum(II) (hereafter called cisplatin) and by other platinum complexes in anticancer chemotherapy, the number of neoplasms that can be succesfully treated with these compounds is still limited. Platinum derivatives are in fact scarcely active against several malignancies with high social incidence, such as non-small cell lung carcinomas, lung adenocarcinomas and adenocarcinomas of the colon and rectum [1-4]. As a consequence, there is a considerable interest in investigating compounds of other transition metals as potential antineoplastic agents [5-8]. The aim of this reasearch is that of finding new active derivatives with a spectrum of action different from that of cisplatin and, if possible, with a minor host toxicity. Many different classes of coordination compounds and organometallic derivatives have been screened on model tumors with this aim, and some promising results have been obtained with derivatives of different metals, e.g. Ga [9,10], Sn [11-13], Rh [14], Au [15], Ti [16-19] and Ru. In this paper we will focus on ruthenium derivatives.

The antitumor properties of simple chloro-ammino-ruthenium(III) derivatives were first investigated by M.J. Clarke [20-25]. Two complexes of this class, namely *cis*-$[Ru(NH_3)_4Cl_2]Cl$ and *fac*-$Ru(NH_3)_3Cl_3$ (Figure 1), showed a significant activity against P388 leukemia, reaching T/C values of 154 and 189, respectively. In more recent years Keppler and coworkers reported that anionic Ru(III) complexes with eterocyclic nitrogen ligands (L) of general formula $(LH)_2[RuCl_5L]$ [26] and (LH)[*trans*-$RuCl_4L_2$] [27] have good antitumor activity against several screening tumor lines [28-31]. In particular, two monoanionic complexes, ImH[*trans*-$RuCl_4(Im)_2$] (**ICR**) (Im = Imidazole) (Figure 1) and the analogous indazole (Ind) derivative, were shown to possess good activity against the platinum resistant chemically-induced colorectal tumors in rats.

Figure 1. Some antitumor active ruthenium(III) complexes: **a)** *fac*-$RuCl_3(NH_3)_3$; **b)** *cis*-$[RuCl_2(NH_3)_4]Cl$; **c)** (ImH)[*trans*-$RuCl_4(Im)_2$] (**ICR**).

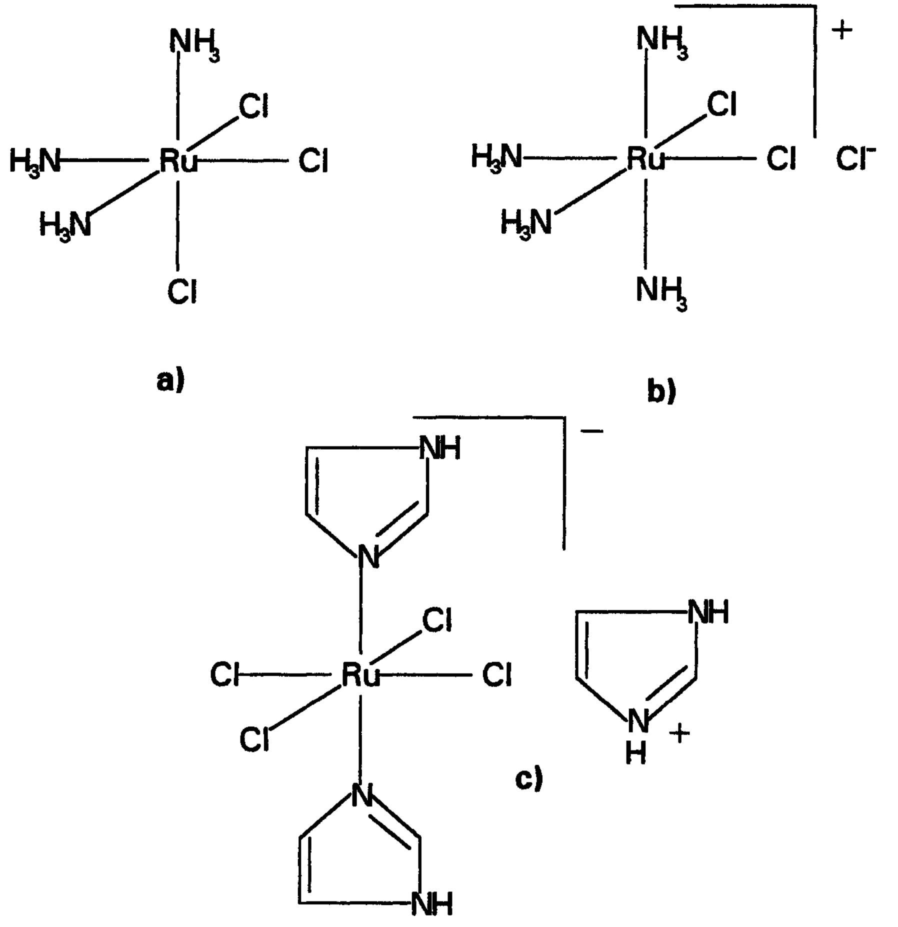

In this tumor model, whose sensitivity to chemotherapeutic agents is virtually the same as that of its human counterpart, the ruthenium derivatives resulted considerably more active in reducing the tumor mass than the clinically used drug, 5-fluorouracil. No hypothesis was advanced on the mechanism of action of such complexes, which are now in advanced preclinical stage [31]. An "activation by reduction" mechanism was instead proposed [20-25] to explain the activity of the chloro-ammino ruthenium derivatives that are considerably inert toward ligand loss opening up coordination positions. According to this hypothesis the inert, and therefore inactive, Ru(III) complexes are considered as prodrugs that can be activated by an in situ reduction to the corresponding more labile Ru(II) species. These should be able to form covalent bonds with biological targets after relatively rapid dissociation of some ligands. A biologically accessible redox potential is obviously required for a complex in order to fit in such mechanism. A higher Ru(II)/Ru(III) ratio might be expected in solid tumor tissues, that are generally considered as reducing, hypoxic environments compared to surrounding, more aerated tissues [32,33]. This feature would promote accumulation of ruthenium in tumor masses. Under such hypothesis the selective cytotoxicity of a complex would depend, beside on other properties such as net charge and liposolubility, on the Ru(III)/Ru(II) redox potential and on the rate of electron transfer. It should be noted, however, that even though Ru(III) species are usually more inert than the corresponding Ru(II) derivatives, the rate of ligand dissociation strongly depends on the nature of the ligands themselves. For example, the presence of trans-labilizing ligands can considerably enhance the dissociation rates. Ru(III) complexes with such features might directly interact with the biological targets.

The *in vivo* distribution of ruthenium, due to its similarities with iron, might be further affected by interactions with the Fe-transport protein, transferrin [24,25,31,35]. Ru(III) has indeed a high affinity for the transferrin Fe-binding sites, and the protein might play an important role in the cellular uptake mechanism of ruthenium [31,34,35]. In such hypothesis, an accumulation of ruthenium in tumor masses might be expected, owing to the high iron requirement of rapidly growing tumor tissues that involves a large number of transferrin receptors.

Our group has been investigating the biological properties of ruthenium-sulfoxide complexes since the late seventies. Dimethylsulfoxide (DMSO), like the other sulfoxides of general formula $R_1R_2SO$, is an ambidentate ligand (Figure 2), as it can coordinate to a metal center either through the sulfur atom (DM*S*O) or through the oxygen atom (DMS*O*) [36,37].

Figure 2. The two main binding modes of dimethyl sulfoxide.

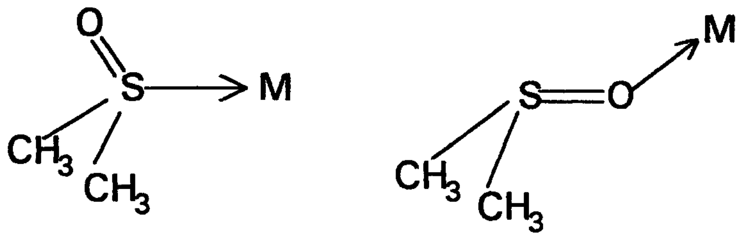

The binding mode depends both on electronic and steric factors. When S-bonded, DMSO acts also as a π-acceptor of electron density and exerts a considerable *trans*-labilizing effect. As a π-acceptor, DMSO stabilizes metal ions in low oxidation states; accordingly, the reduction potential of a DMSO complex may be tuned by the number and binding mode (S- or O-bonding) of coordinated sulfoxides. Finally, dimethylsulfoxide is known to diffuse very easily through biological membranes and might improve the diffusion of coordinated metal ions as well.

The work of our group began with the investigation of Ru(II)-DMSO complexes, in particular *cis*- and *trans*-$RuCl_2(DMSO)_4$ [38-48], and more recently focused on Ru(III) derivatives [49-56]. In this paper we will report a survey of our latest results of ruthenium(III)-sulfoxide complexes from three different, but strictly related, points of view: structure and chemical behavior, antitumor activity and interactions with DNA, considered as one of the possible molecular targets.

### Results and Discussion

**Chemical Aspects.** The precursors of the compounds subject of this study are $(DMSO)_2H$[*trans*-$RuCl_4(DMSO)_2$] (**1**) and its sodium salt and *mer*-$RuCl_3(DMSO)_3$ (**2**).

Figure 3. Schematic synthetic pathways to **1** and **2**.

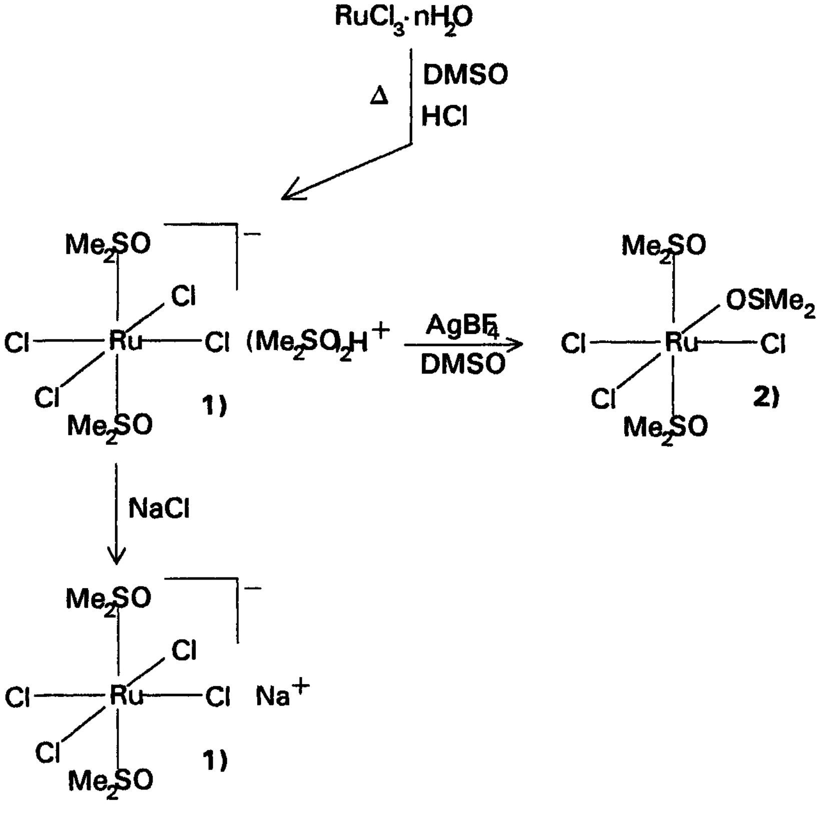

The synthetic pathways to **1** and **2** and their structural features [51] are schematically reported in Figure 3. A completely analogous scheme might be drawn for the corresponding tetramethylenesulfoxide (TMSO) derivatives **3** and **4** [49].

The ruthenium(III)-sulfoxide complexes **1-4**, being structurally very similar to the Ru(III) compounds with heterocyclic nitrogen donor ligands reported by Keppler [26,27], appeared particularly attractive in the perspective of finding new derivatives to be tested for antitumor activity. In fact, the anionic compounds are isostructural and isoelectronic to **ICR**, with S-bonded sulfoxides replacing the nitrogen ligands. Unlike **ICR**, however, the ruthenium-sulfoxide complexes proved to be rather labile in aqueous solution, in particular at physiological pH where they are readily hydrolized [51].

Although complexes **1-4** resulted scarcely attractive for pharmacological development due to their lability, the investigation of their reactivity led us to observe that one of the two trans S-bonded sulfoxides could be easily substituted by a nitrogen ligand [55].

According to this reactivity, they become the precursors of two new classes of ruthenium(III) compounds of general formula Na[*trans*-$RuCl_4(R_2SO)(L)$] (**A**) and *mer,cis*-$RuCl_3(R_2SO)(R_2SO)(L)$ (**B**), ($R_2SO$ = DMSO, TMSO), with L = nitrogen donor ligand (Scheme 1-2).

**Scheme 1**

Na[*trans*-$RuCl_4(R_2SO)_2$] + L ---> Na[*trans*-$RuCl_4(R_2SO)(L)$] + $R_2SO$

**Scheme 2**

*mer*-$RuCl_3(R_2SO)_3$ + L ---> *mer,cis*-$RuCl_3(R_2SO)(R_2SO)(L)$ + $R_2SO$

In the case of the anionic derivatives, sodium could be rather easily replaced by other cations such as $NEt_4^+$ and $LH^+$ (Scheme 3):

**Scheme 3**

Na[*trans*-$RuCl_4(R_2SO)(L)$] + $X^+$ ---> X[*trans*-$RuCl_4(R_2SO)(L)$] + $Na^+$

The reaction procedure turned out to be very versatile and, besides the derivative with L = $NH_3$, we prepared derivatives with 5-membered heterocyclic ligands such as imidazole (Im), N-methylimidazole (MeIm), pyrazole (Pz), oxazole (Ox), with 6-membered heterocyclic ligands such as pyridine (Py) and, finally, with condensed heterocylcic rings such as indazole (Ind), isoquinoline (Iq) and 1,5,6-trimethylbenzimidazole ($Me_3Bzm$).

The complexes have been fully characterized by common spectroscopic techniques (UV-vis, IR, NMR) and the crystal structure of some of them was determined [55]. As expected, replacement of one DMSO with an N-donor ligand involved a strengthening of the remaining Ru-DMSO bond, attributable to the diminished competition for $\pi$-back bonding electrons between the two trans ligands. The crystal structure of the imidazole derivative, Na[*trans*-$RuCl_4$(DMSO)(Im)] (**5**), is particularly significant to this regard, as the complex can be considered as a sort of hybrid between the two symmetrically disubstituted derivatives **1** and **ICR** (Figure 4). In complex **5** a remarkable shortening of the Ru-S bond length with respect to **1** [2.296(1) vs 2.348(1) Å] is observed, while the Ru-N distance is practically the same as in **ICR**.

Figure 4. Structural comparison of Na[*trans*-$RuCl_4$(DMSO)(Im)] (from ref. 55) with $(DMSO)_2H$[*trans*-$RuCl_4(DMSO)_2$] (from ref. 51) and (Im)H[*trans*-$RuCl_4(Im)_2$] (**ICR**) (from ref. 27)

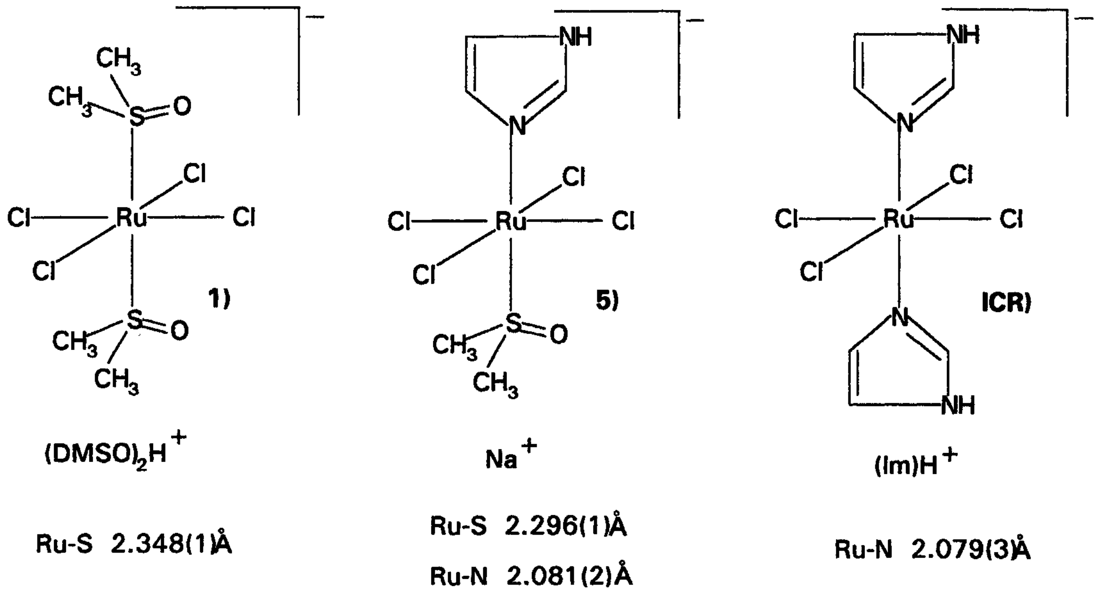

The sodium salts of the anionic derivatives are highly water soluble but the reproducibility of their formulation is sometime affected by their tendency to crystallize including DMSO and acetone molecules of crystallization in variable ratio. Perfectly reproducible elemental analyses are instead obtained with the $LH^+$ and $NEt_4^+$ derivatives, which do not have molecules of crystallization and whose solubility is still high enough for pharmacological tests. The solubility of the neutral complexes is less pronounced, approximately 2 mg/ml for *mer*-$RuCl_3(DMSO)_2(NH_3)$ and *mer*-$RuCl_3(DMSO)_2$(Im). As expected, the liposolubility of the complexes, measured as partition coefficient between water and n-octanol, increases on replacing DMSO with TMSO and on increasing the lipophylicity of the nitrogen ligand (Table 1) [56]. The use of properly substituted ligands might strongly influence this parameter. A higher liposolubility is usually associated with an increased capability of crossing cell membranes through a passive transport mechanism [57].

The redox potential of selected compounds was measured by cyclic voltammetric experiments [55,56] (Table 1). All complexes undergo a reversible uncomplicated reduction, with an observed $E_{1/2}$ that falls in the range -0.001 to +0.117 V (vs SSCE). Neutral derivatives are significantly more reducible than the corresponding anionic species. No apparent changes in the redox potential result from the substitution of a DMSO for a TMSO ligand.

Table 1. Redox potentials, partition coefficients and visible absorption bands of some Ru(III) complexes.

| Complex | $E_{1/2}$ (V vs SSCE)[a] | log P[b] | $\lambda_{max}$(nm) ($\varepsilon$ ($M^{-1}cm^{-1}$)) |
|---|---|---|---|
| (Im)H[*trans*-$RuCl_4(Im)_2$][c] | -0.511 | - | - |
| Na[*trans*-$RuCl_4$(DMSO)($H_2O$)] | 0.107 | - | 464 (592) 395 (5212) |
| Na[*trans*-$RuCl_4$(DMSO)($NH_3$)] | 0.029 | 3.46 | 449 (449) 385 (3684) |
| Na[*trans*-$RuCl_4$(DMSO)(Im)] | -0.001 | 3.79 | 451 (488) 390 (3644) |
| Na[*trans*-$RuCl_4$(DMSO)(Pz)] | 0.065 | - | 456 (673) 389 (4776) |
| Na[*trans*-$RuCl_4$(DMSO)(Ind)] | 0.089 | 2.63 | 461 (648) 395 (4357) |
| Na[*trans*-$RuCl_4$(DMSO)(Py)] | 0.065 | 3.21 | 458 (438) 394 (3568) |
| Na[*trans*-$RuCl_4$(DMSO)(Iq)] | 0.066 | 2.30 | 458 (488) 395 (3850) |
| Na[*trans*-$RuCl_4$(TMSO)(Im)] | - | 3.21 | 451 (496) 388 (3411) |
| Na[*trans*-$RuCl_4$(TMSO)(Py)] | 0.062 | 2.80 | 457 (474) 394 (3539) |
| Na[*trans*-$RuCl_4$(TMSO)(Iq)] | - | 1.91 | 457 (492) 395 (3497) |
| $RuCl_3(DMSO)_2(H_2O)$ | 0.186 | - | 426 (1060) 364 (3260) |
| $RuCl_3(DMSO)_2(NH_3)$ | 0.117 | 1.00 | 414 (1174) 355 (3222) |
| $RuCl_3(DMSO)_2$(Im) | 0.101 | 0.82 | 413 (1034) 358 (2529) |

[a] $E_{1/2}$ obtained at Pt electrode; SSCE = saturated calomel electrode. [b] P, partition coefficient =(concentration in water)/(concentration in n-octanol). [c] $E_{1/2}$ obtained at Hg electrode; the signal is reversible only in the presence of excess imidazole ([Im]/[ICR] = 120).

According to the measured $E_{1/2}$, a biological reduction process is thermodynamically possible for all the derivatives [24]. In this respect it is interesting to compare the $E_{1/2}$ value of **5** with that of **ICR**, which we found to be -0.511 V under the same conditions. This last value sits at the lower limit of the biologically accessible potentials suggesting that, in the hypothesis of an "activation by reduction" mechanism operating *in vivo* for these Ru(III) complexes, the sulfoxide derivatives might be expected to behave quite differently from **ICR** despite their structural similarity.

Due to their greater solubility in water, anionic complexes of class **A** are more attractive for being developed as drugs. Accordingly, most of the pharmacological and chemical investigations to date have been focused on this class of compounds. The chemical behavior of the anionic complexes has been studied both in unbuffered distilled water and in phosphate-buffered solutions at physiological pH. The stability of the complexes in the slightly acidic distilled water can give valuable informations concerning the administration of the product, while the behavior under physiological conditions is strictly related to the mechanism of action of the complexes and their *in vivo* distribution.

**a) Distilled water.** The evolution with time of the complexes has been followed by visible spectroscopy and $^1H$ NMR. All complexes behave similarly and are quite inert in distilled water at 25°C, as clearly shown by the rather slow spectral changes with time. Each complex of class **A** is characterized by a strong charge-transfer absorption band around 390 nm (Table 1) whose intensity slowly decreases with time upon dissolution in water (12hr $< t_{1/2} <$ 24hr, depending on the nature of the nitrogen ligand). No new spectral feature appears and the process is accompanied by an increase of diffused background absorption and a decrease of pH, attributed to the formation of poly-oxo or poly-hydroxo species [58] as for complexes **1-4**. The NMR spectra deserve some comments, being particularly helpful in determining the chemical behavior of the complexes in solution. Owing to the paramagnetism of the Ru(III) nucleus, $^1H$ NMR spectra give rather broad peaks dispersed over a wide range of chemical shift. The $D_2O$ $^1H$ NMR spectra of the anionic complexes **A** share the common feature of a broad signal of S-bonded DMSO centered at about -15 ppm [55]. Analysis of the neutral complexes **B** was limited to the ammonia and imidazole derivatives, due to solubility limits. Similarly to the precursor [51], they both have a broad resonance around -14.5 ppm (S-bonded DMSO) and a slightly sharper signal of equal intensity around 10.7 ppm (O-bonded DMSO). Owing to their lower intensity, detection of the resonances of the nitrogen ligand was sometimes difficult, in particular with the slightly soluble neutral derivatives. Accordingly, due also to the almost total absence of previous reports on this subject, a complete assignement of the NMR spectra was not attempted. As a general remark, the NMR spectra of complexes **A** and **B** clearly showed that neither DMSO or the nitrogen ligand are readily replaced in aqueous solution. In the anionic derivatives DMSO slowly dissociates with time, as shown by the increase of the signal of the free ligand at 2.71 ppm. The amount of DMSO dissociation is between 25 and 50% after 24hr, depending on the nature of the nitrogen ligand. Dissociation of this latter is remarkably slower. The inertness of the complexes can be further increased by working at lower pH, such as in pH 4 acetate buffer, or in the presence of small amounts of DMSO.

**b) Physiological pH.** Due to the "activation by reduction" hypothesis proposed for Ru(III) species [20-25], we devoted a particular attention to the effect of biological reductants on the chemical behavior of the complexes. We found that, at 25°C in pH 7.4 phosphate buffer, the anionic complexes are very rapidly reduced by stoichiometric amounts of monoelectronic biological reductants, such as ascorbic acid, glutathion and cystein. The reduction can be followed spectroscopically in the visible, since in the process the solution becomes almost colorless. On the contrary, the reduction with bielectronic reductants, such as glucose or lactic acid, even though thermodynamically very favourable, does not occurr. It is worth noting that, under the same experimental conditions, **ICR** is unaffected by the presence of either type of reductant. Since the reduction is much faster than any substitution process, an outer sphere reduction mechanism can be hypothized. In agreement with the cyclic voltammetry experiments, the dianionic complex reported in Scheme 4 is very likely the first species formed upon reduction.

Scheme 4. Behavior of [*trans*-$RuCl_4(DMSO)(L)]^-$ complexes in the presence of stoichiometric amounts of monoelectronic reductants.

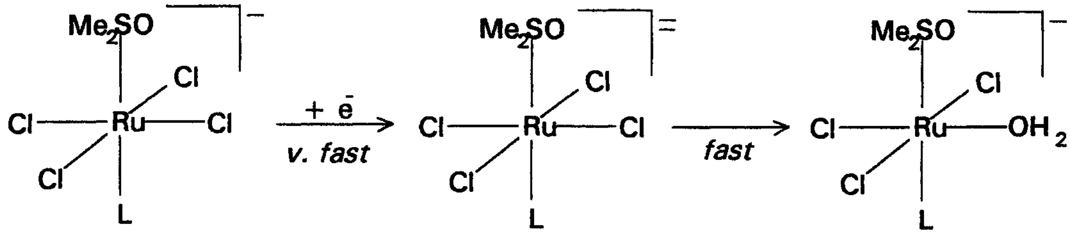

According to the reactivity of anionic ruthenium(II)-chloride-DMSO complexes [59], a rather fast chloride dissociation from this species can be reasonably envisaged. Further chloride dissociation and/or reaction with the reducing agent can not be excluded.

In pure buffered solutions we observed that, upon increasing the pH, the anionic complexes become increasingly more labile. Three, quite well separated, consecutive steps can be distinguished at pH 7.4 (25 °C) and separately followed by visible and NMR spectroscopy. The visible spectral changes occurring during the first step, which lasts approximately 1 hour at 25 °C, are represented in Figure 5. The absorption pattern of the starting complex decreases with an autocatalytic kinetic profile and the spectrum of the species that builds up, with a maximum around 346 nm, is very similar to those of the neutral derivatives **B**. A neat isosbestic point, showing the presence of only two species in solution, is maintained during the process. According to this pattern, dissociation of a chloride was the most likely process occurring in step 1. This hypothesis was confirmed by NMR data since, during step 1, the original signals of S-bonded DMSO and of the nitrogen ligand in **A** are gradually replaced by new signals, still attributable to bound ligands.

Figure 5. Visible spectral changes observed in step 1 for Na[*trans*-$RuCl_4$(DMSO)(Im)] (2 x $10^{-4}$ M; 60 mM phosphate buffer pH = 7.4; T = 25 °C; scan time interval 5 min).

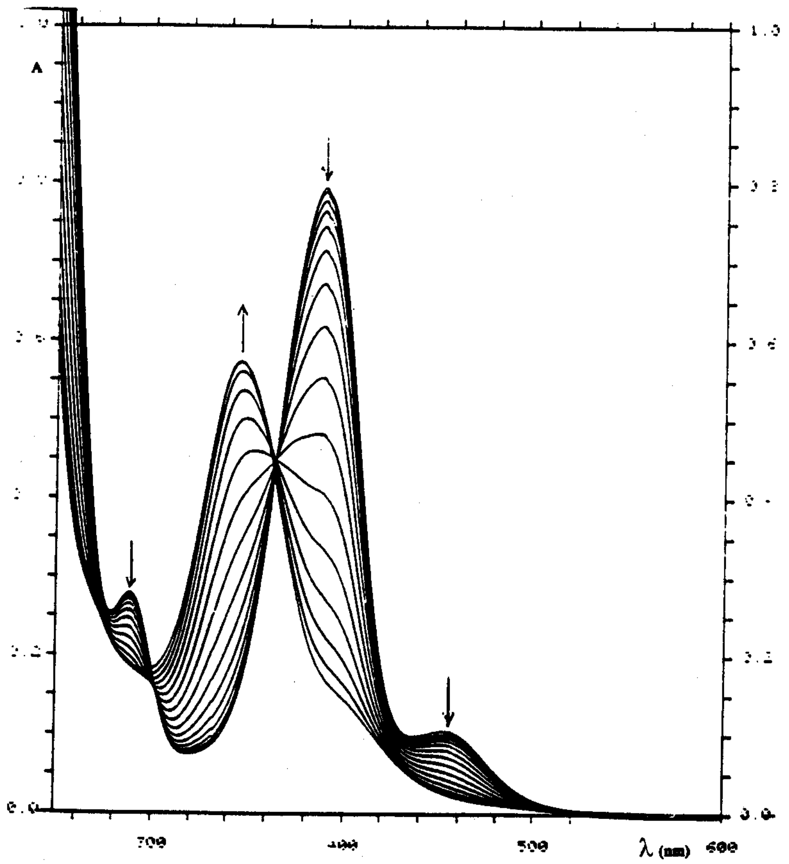

The case of the N-methylimidazole derivative is particularly significative to this regard and is reported in Figure 6. Upon chloride dissociation, the broad resonance of DMSO moves from -15 ppm to approx -11 ppm, while the sharper resonance of N-$CH_3$ moves from -1 ppm to +1 ppm. Only one signal for the imidazole protons could be detected (it moves from -3.8 ppm to -0.5 ppm during step 1), the others being very likely too broad to be detected. Only a small amount of free DMSO (6%) could be found at the end of step 1. The reaction rate of this step increases along with the pH.

Figure 6. Changes observed in the $^1H$ NMR spectrum of Na[*trans*-$RuCl_4$(DMSO)(MeIm)] during step 1. Only the most relevant part of the spectrum is shown.

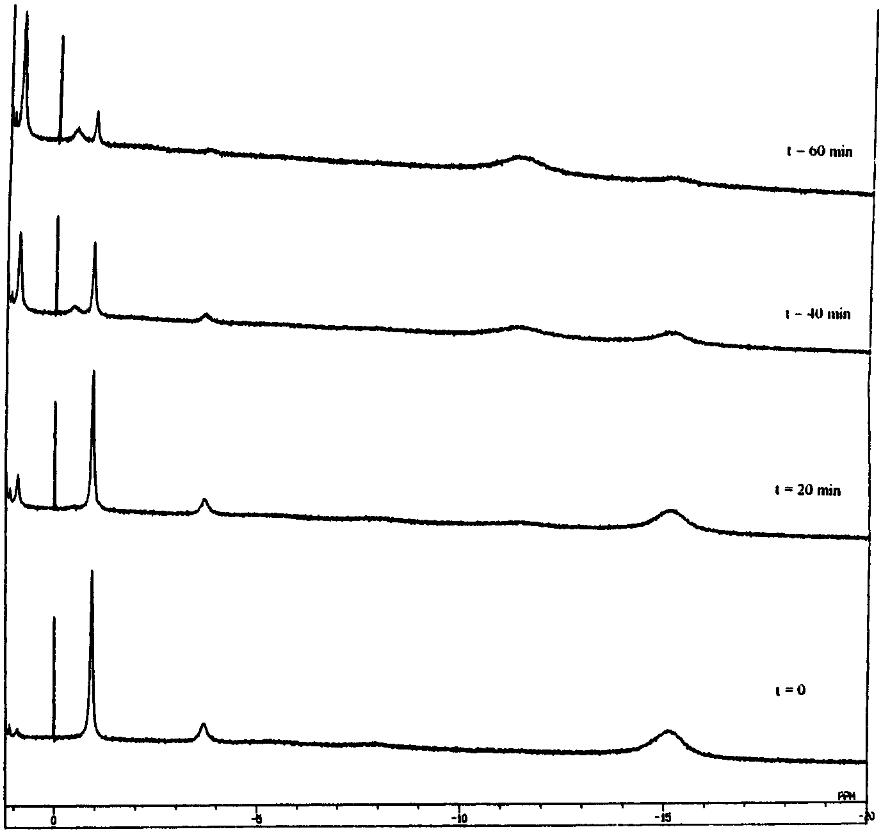

We also found that, in the presence of catalytic amounts of reducing agents, the reaction rate remarkably increases and the kinetic profile becomes of pseudo-first order (Figure 7). These data suggest that chloride substitution is catalyzed by traces of Ru(II) species; similar processes have already been described for inert Ru(III) species [20]. In the absence of added reductants, we hypothize that an autoreduction process, parallel to the substitution reaction, is the source of Ru(II) species. A partial reduction due to trace impurities in solution would not explain the autocatalytic pattern, since in that case the amount of catalyst would be constant since the beginning.

The presence of traces of added biological reductants promotes the formation of an equivalent amount of catalyst, so that the reaction proceeds mainly through the catalyzed path and becomes zero order in the catalyst and first order in the reactant.

In the second step of the reaction the product that built up in step 1 is transformed into a new species. This process involves the decrease of the absorption maximum at approx 346 nm, with formation of a new maximum at higher frequencies (325 nm) and a concomitant gradual increase of the background absorption (Figure 8). An isosbestic point is still maintained.

Figure 7. Visible spectral changes observed in step 1 for $[NEt_4]$[*trans*-$RuCl_4$(DMSO)(Im)] in the presence of 1% glutathion (GSH) ([Ru] = 2 x $10^{-4}$ M; [GSH] = 2 x $10^{-6}$ M; 60 mM phosphate buffer pH = 7.4; T = 25 °C; scan time interval 5 min) .

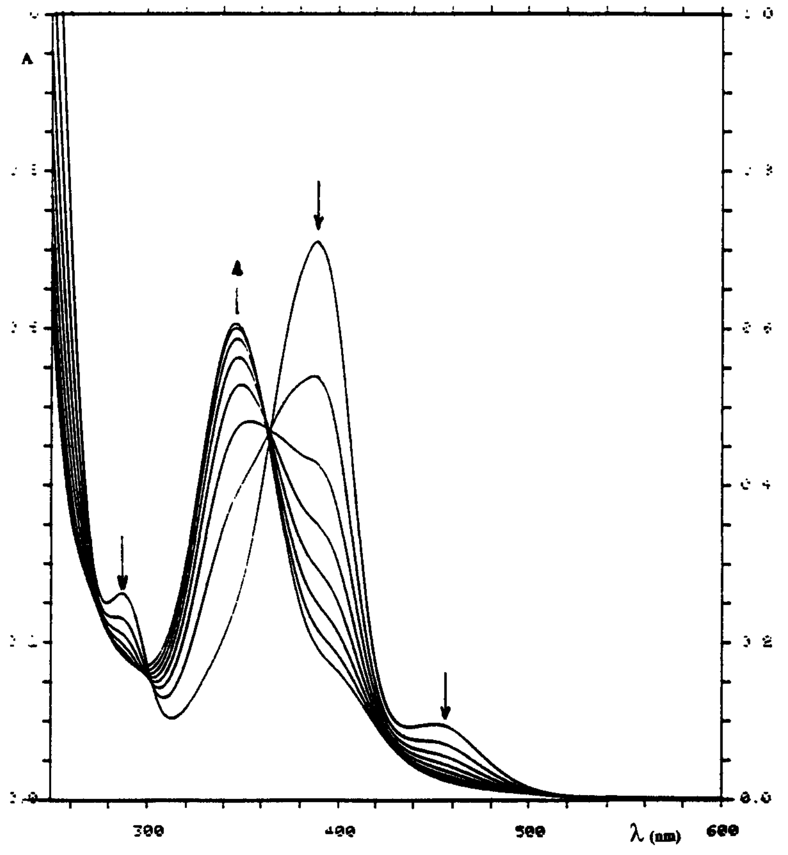

Step 2 is independent on the concentration of $OH^-$ and, unlike step 1, its rate is not influenced by the presence of trace reductants. However, stoichiometric amounts of reductants introduced in solution at this stage determine complete reduction to Ru(II) species.

In agreement with the blue-shift observed in the visible spectrum, step 2 has been tentatively attributed to the dissociation of a further chloride but, according to the NMR spectra, it is also accompanied by the partial dissociation of DMSO and of the nitrogen ligand. However, since no new signals for bound ligands appear in the NMR pattern during this step, formation of a dimeric species might be also hypothized. In this hypothesis the presence of two paramagnetic centers would broaden the signals of coordinated ligands to such an extent as to make them undetectable.

The following step 3 is accompanied by a general increase of the background absorption, attributed to the formation of polymeric species. The chemical behavior at physiological pH is summarized in Scheme 5, where the likely deprotonation equilibria of the aquo species have been also evidenced.

Figure 8. Visible spectral changes observed in step 2 for Na[*trans*-$RuCl_4$(DMSO)(Im)] (2 x $10^{-4}$ M; 60 mM phosphate buffer pH = 7.4; T = 25 °C; scan time interval 10 min).

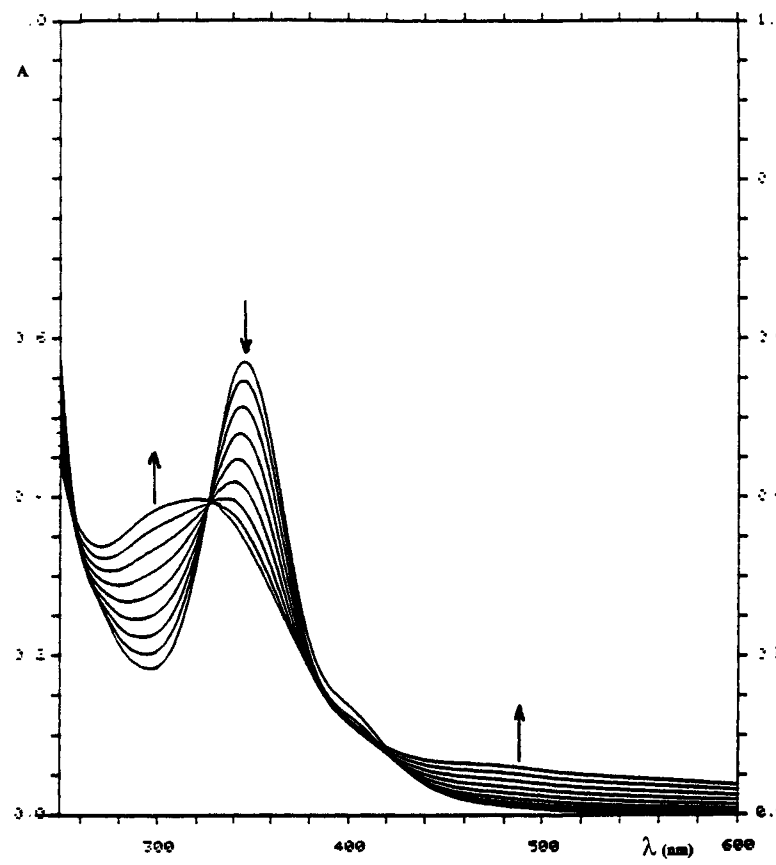

As a general conclusive remark, we can state that, owing to the reinforcement of the Ru-S bond, all complexes of class A are indeed remarkably more inert than their precursors in aqueous solution at physiological pH.

We also investigated the behavior of the complexes in the presence of a slight excess of a nitrogen ligand, such as imidazole (Im/Ru = 10), in order to mimic the presence of the biological targets. Step 1 and 2 are unaffected by the nitrogen ligand, while in the last step the backgroud absorption increase becomes almost negligible and a new species with an absorption maximum around 290 nm forms.

This behavior suggests that **i)** the complexes are not reactive as such towards the biological targets but must first undergo a series of chemical transformations; **ii)** in a biological environment, due to the abundance of possible reactants, the formation of polymeric species should be prevented. Moreover, due to the aboundance of reductants in biological systems, the complexes might become reduced before undergoing any dissociation process. For the sake of comparison, we found that **ICR** is considerably more inert compared to the sulfoxide derivatives of class **A** also at physiological pH and it is not affected by reductants.

Scheme 5. Chemical behavior of the complexes of class A at physiological pH

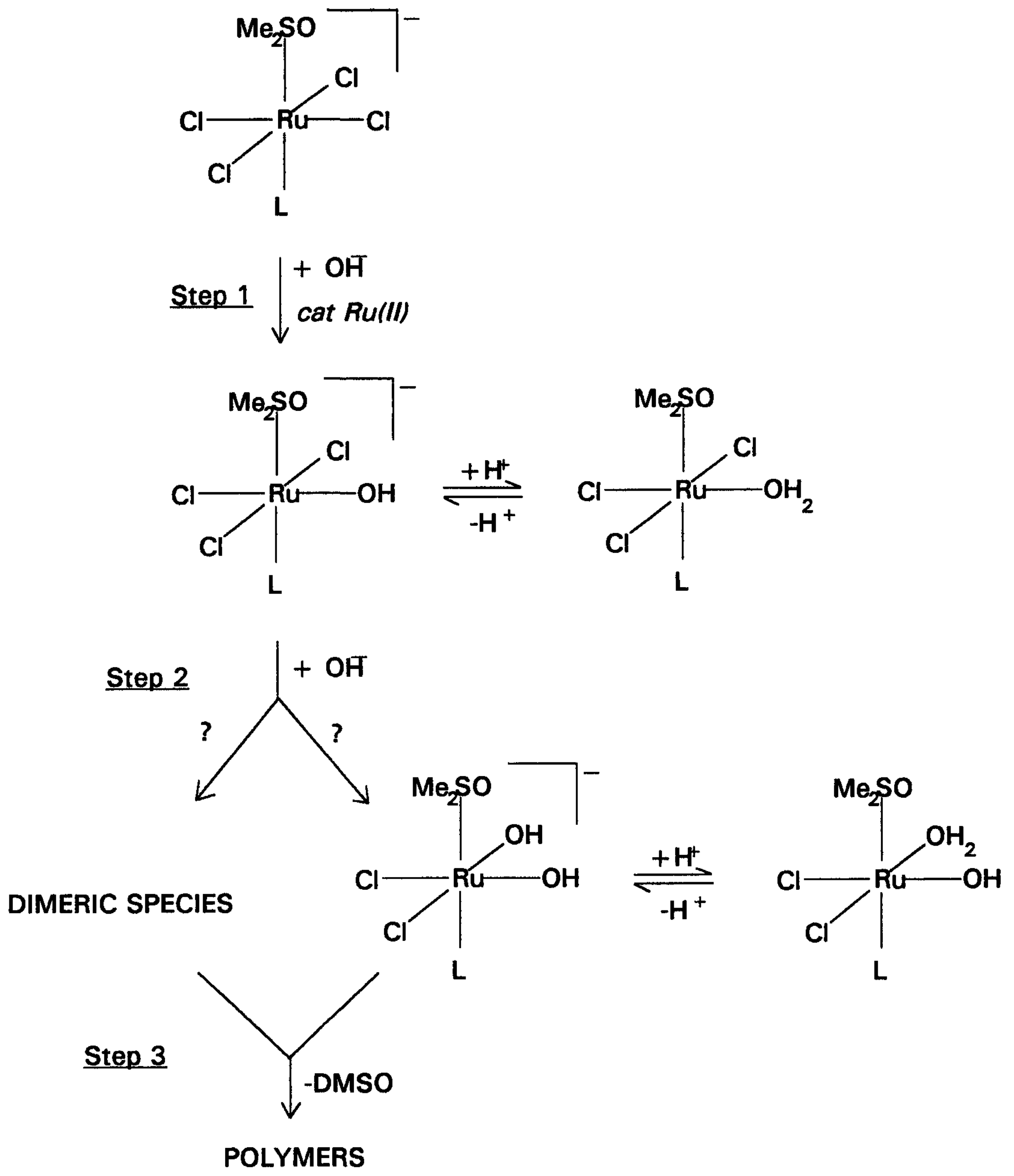

**Pharmacology.** The investigations aimed to evaluate the antitumor activity of the above compounds were carried out with tumors such as Lewis lung carcinoma, B16 melanoma, TLX5 lymphoma, P388 lymphocytic leukemia and MCa mammary carcinoma. However, a large part of the characterization of the effects of ruthenium(III)-dimethylsulfoxide complexes on metastasizing tumors has been done on MCa mammary carcinoma. This tumor, similarly to almost all solid metastasizing tumors, after s.c. or i.m. implantation produces distant metastases (to the lungs) and therefore appears to be suitable for studying anticancer agents active on the disseminated tumor.

The results of the differential effects of a series of class A ruthenium complexes on primary tumor growth and on the formation of spontaneous pulmonary metastases in the model of MCa mammary carcinoma of CBA mouse are reported in Table 2.

Table 2. Differential effects of some complexes on primary tumor growth and on lung metastasis formation.

| Compound | Dose | Primary tumor | Lung metastases | |
|---|---|---|---|---|
| | mg/Kg/day | weight (g) | number | weight (mg) |
| Controls | - | 1948±151 | 31.0±2.0 | 14.6±2.4 |
| | 100 | 1060±194* | 11.2^ | 0.04 |
| Na[*trans*-$RuCl_4$(DMSO)Im] | 50 | 1644±138 | 18.7±3.7* | 0.6±0.3** |
| | 25 | 1687±227 | 35.0±13.1 | 7.3±5.2* |
| | 90 | 1606±221 | 6.0±2.0** | 0.9±0.3** |
| Na[*trans*-$RuCl_4$(DMSO)Ind] | 45 | 2079±152 | 8.3±2.3** | 2.0±0.8** |
| | 22.5 | 1738±172 | 13.2±2.5* | 8.1±2.2* |
| | 30 | 2079±240 | 13.7±3.3* | 7.0±2.2** |
| Na[*trans*-$RuCl_4$(TMSO)Iq] | 15 | 2348±122 | 11.2±2.2* | 3.8±0.7** |
| | 7.5 | 1931±189 | 16.3±3.5* | 10.9±2.2 |
| | 90 | 766± 77** | 1^ | 0.13 |
| Na[*trans*-$RuCl_4$(DMSO)Ox] | 45 | 1282± 80** | 3.6±0.4** | 0.4±0.1** |
| | 22.5 | 2086±195 | 19.0±4.7* | 6.0±2.6** |

: 6/8 animals free of macroscopically detectable metastases; * and ** mean statistically different from controls at $p < 0.05$ and $p < 0.01$ level, respectively (Student-Newman-Keuls analysis of variance).
Groups of 8 mice, implanted i.m. with $10^6$ MCa mammary carcinoma cells on day 0, were given i.p. the test compounds on days 1,5,9,13. Primary tumor weight was determined on day 14 and lung metasases were counted on day 21.

From these studies it is evident how, independently on the compound being used, the effects on lung metastasis formation are always higher than those on primary tumor growth. Interestingly, Na[*trans*-$RuCl_4$(TMSO)Iq] that, given the significant cytotoxicity for tumor cells evidenced *in vitro* against TLX5 lymphoma cells (Table 3), was expected to give the higher reduction of primary tumor growth, resulted instead endowed with a rather weak activity against tumor growth and lung metastases; similarly it resulted scarcely active on TLX5 lymphoma or on P388 leukemia, including the cisplatin resistant subline, after *in vivo* treatments (Table 4). Conversely, compounds such as Na[*trans*-$RuCl_4$(DMSO)Im] or Na[*trans*-$RuCl_4$(DMSO)Ox], completely devoid of *in vitro*

cytotoxicity, significantly reduced primary tumor growth and gave an even more pronounced reduction of the formation of spontaneous pulmonary metastases in the same animals. These effects have been obtained at doses that are equitoxic for the host in that cause the same effect on body weight gain during treatment.

Table 3. Effects of some complexes on TLX5 lymphoma *in vitro*.

| Compound | Optimal ILS% | $DE_{50}$ | Global assesment |
|---|---|---|---|
| Na[*trans*-$RuCl_4$(DMSO)Im] | +7.4 | $>>10^{-3}$M | - |
| Na[*trans*-$RuCl_4$(DMSO)Ox] | +8.4 | $>>10^{-3}$M | - |
| Na[*trans*-$RuCl_4$(DMSO)Ind] | +24 | $>10^{-3}$M | + |
| Na[*trans*-$RuCl_4$(DMSO)Iq] | +32 | $>10^{-3}$M | ++ |
| Na[*trans*-$RuCl_4$(TMSO)Iq] | 100% cured | $10^{-4}>10^{-5}$M | ++++ |

Optimal ILS%: optimal increase of survival time over untreated controls; $DE_{50}$: Molar concentration that increses by 50% the life span of the transplanted mice; Global assesment: - and + indicate the relative potency of the compounds; -=inactive.

Groups of 5 CBA male mice, transplanted i.p. with aliquots of 0.1 ml of an incubation mixture containing $10^6$ TLX5 lymphoma cells per ml kept *in vitro* at 37°C for 60 min in the presence of $10^{-5}$, $10^{-4}$ or $10^{-3}$M concentrations of each compound, were evaluated for tumor development and survival time. 100% cured means that no transplanted animal developed tumor within 90 days.

Anyhow, it must be stressed that, although the activity on tumors of lymphoproliferative type such as the P388 lymphocytic leukemia are not much impressive (Table 4), all compounds tested were active on the cisplatin resistant line, in some cases with an activity clearer than that observed on the parental line, thus indicating that ruthenium-dimethylsulfoxide complexes do not exibit cross resistance with cisplatin itself.

Table 4. Effects of some complexes on TLX5 lymphoma and on P388 or P388/DDP leukemias *in vivo*.

| Compound | TLX5 lymphoma | P388 | P388/DDP |
|---|---|---|---|
| Na[trans-$RuCl_4$(DMSO)Im] | nt | 170 [40] | 162 [10] |
| Na[trans-$RuCl_4$(DMSO)Ind] | 120 [20] | 156 [20] | 152 [20] |
| Na[trans-$RuCl_4$(DMSO)Iq] | 122 [20] | 168 [40] | 162 [20] |
| Na[trans-$RuCl_4$(TMSO)Iq] | 124 [10] | 121 [10] | 139 [10] |

Each value is the mean %T/C obtained at the optimal dose [in brackets as mg/Kg/day] with groups of 5 mice treated i.p. daily on days 1-7 from i.p. tumor implantation of $10^5$ TLX5 lymphoma cells or of $10^6$ cells of P388 leukemia or of its cisplatin resistant line.

Out of the compounds studied so far, only Na[*trans*-RuCl4(DMSO)Im] (5) exibited an interesting relationship between inhibition of primary tumor growth and increase of survival time of the treated hosts, with a global efficacy independent on the tumor line being used. Complex 5 reduces the growth of solid tumors implanted i.m. or s.c. [see also data from ref. 53,54] and parallely increases the survival time of the same animals also when the effects on primary tumor growth are not as pronounced as one would expect to account for the increased life-span. In this context, it must be emphasized that cisplatin, even when capable of reducing i.m. tumor growth of MCa mammary carcinoma by more than 90% (as resulting from a separate experiment designed as to get primary tumors at 2-weeks of less than 0.5 g) was practically inactive on host survival time (T/C= 121±20%). The effects of **5** on survival time could be explained, if not completely at least in part, by its potent effectiveness in reducing lung metastasis formation, as results from studies on lung metastasis either spontaneously formed from i.m. tumor implants or artificially obtained by i.v. implantation of tumor cells (Table 5). The antimetastatic effects of **5** depend on the treatment schedule chosen and, similarly to what observed on primary tumors, are more pronounced with low doses given daily rather than with large doses given with drug-free intervals (see also data from ref. 54). On the contrary, the activity on metastases of its analog Na[*trans*-$RuCl_4$(TMSO)Iq] is less pronounced. These data further stress the absence of any correlation between *in vitro* cytotoxicity and *in vivo* antitumor activity and seem to suggest that the increase of lypophylicity of the complexes from logP= 3.79 of Na[*trans*-$RuCl_4$(DMSO)Im] to logP= 1.91 of Na[*trans*-$RuCl_4$(TMSO)Iq] (Table 1) does not produce benefits to the antimetastatic properties.

Table 5. Effects of Na[*trans*-$RuCl_4$(DMSO)Im] and of Na[*trans*-$RuCl_4$(TMSO)Iq] on spontaneous metastases and on lung colonies.

| Compound | Spontaneous metastases | | Lung colonies | |
|---|---|---|---|---|
| | Number | Weight | Number | Weight |
| Controls | 17.4±5.5 | 8.1±3.6 | 21.4±2.8 | 30.5±7.3 |
| Na[*trans*-$RuCl_4$(DMSO)Im] 44 mg/Kg/day | 3.5±1.0 | 0.8±0.5 | 12.7±2.6 | 2.9±0.6 |
| Na[*trans*-$RuCl_4$(TMSO)Iq] 20 mg/Kg/day | 11.7±3.2 | 4.1±0.8 | 17.0±2.9 | 14.7±3.8 |

Groups of 8 CBA female mice, implanted s.c. with $2x10^6$ MCa mammary carcinoma cells (spontaneous metastases) or i.v. with $10^5$ MCa MCa mammary carcinoma cells (lung colonies) on day 0, were given i.p. the test compounds on days 1-6 and were killed on day 21 or on day 18 for the evaluation of spontaneous metastases or of lung colonies, respectively.

However, data from Table 5 further indicate that spontaneous metastases are a target better than lung colonies artificially obtained by i.v. implantation of tumor cells. Thus, Na[*trans*-$RuCl_4$(DMSO)Im] is not simply effective on tumors growing in the lungs because of its possible higher concentration in the lung tissue, as suggested by a pilot study performed by means of atomic absorption measurements [60], but probably also because on spontaneous lung metastases it can overcome the problems of chemosensitivity usually found within tumor cells of primary tumors, which can also be present on artificial metastases. This behaviour can be explained taking into account the differences in growth kinetics between spontaneous and artificially induced lung metastases, where the latter have been described to show marked similarities with the primary tumor from which they arose [61,62]. Therefore, the selectivity of the effects of Na[*trans*-$RuCl_4$(DMSO)Im] for lung tumors, unlike cisplatin that exerts its antitumor effects on the primary tumor, seems to be attributable to the capacity of **5** to distinguish the differences between tumor cell populations and to be more effective on those with higher metastatic potential. Interestingly, at the lung level, Na[*trans*-$RuCl_4$(DMSO)Im] has no evidence of toxicity for healthy lung epithelium, as results from histological analysis.

Despite the apparent lack of cytotoxic effects for tumor cells or for cells of healthy tissues, Na[*trans*-$RuCl_4$(DMSO)Im] and the related complexes of class **A** are able to interact with DNA *in vitro*, binding to nucleobases and also causing interstrand cross-links that in some cases are of the same magnitude as those caused by cisplatin under the same conditions (Table 6).

The sequence specificity of DNA modification by ruthenium complexes was investigated by the primer extension footprinting technique. DNA from plasmid pBR322 was modified with class A complexes, and with **ICR** and cisplatin for comparison, by *in vitro* treatment at 37°C for 1hr, according to standard methods. The DNA was primed with Pst(+) primer and used as a template for second strand synthesis by Sequenase 2 enzyme. The ruthenium(III)-sulfoxide complexes block replication, as evidenced by the stop sites observed on sequencing gel; on the contrary **ICR** produces no stop sites. All class **A** complexes investigated so far have the same pattern of blocking lesions, showing faint stop bands corresponding to nearly every nucleotide, with the exception of thymine, and more intense stop bands corresponding to guanines. This behaviour could reflect a high capability of class **A** complexes (probably in an hydrolized form) to attack DNA and, consequently, a great tendency of the polymerase to be dissociated from the treated template.

Table 6. Effects of some ruthenium(III) complexes on the % of interstrand cross-linked DNA.

| Compound | Drug/Nucleotide concentration ration ($x10^{-2}$) | | | |
|---|---|---|---|---|
| | 0.25 | 0.5 | 1 | 2 |
| | | | | |
| Cisplatin | 4.04±0.28 | 8.01±0.69 | 15.4±1.12 | 29.0±0.14 |
| ICR | 0 | 0 | 0 | 0 |
| Na[*trans*-$RuCl_4$(DMSO)Im] | 0.34±0.08 | 0.82±0.35 | 2.20±0.41 | 7.38±0.45 |
| Na[*trans*-$RuCl_4$(DMSO)Ind] | 1.46±0.48 | 3.42±0.55 | 7.23±1.35 | 14.9±0.28 |
| Na[*trans*-$RuCl_4$(TMSO)Iq] | 1.57±0.26 | 3.39±1.18 | 10.7±0.41 | 23.9±1.11 |
| Na[*trans*-$RuCl_4$(DMSO)Ox] | 3.33±0.31 | 6.04±2.85 | 12.7±0.77 | 24.8±1.12 |

*In vitro* incubations were made at 37°C for 60 min, and the % of interstand cross-links was determined by meand of the ethidium bromide fluorescence assay, by a modification of the method of Morgan and Pulleybank [63] and Brent [64].

Unlike the above ruthenium(III)-sulfoxide complexes, some ruthenium(II)-sulfoxides derivatives such as *trans*-$RuCl_2(DMSO)_4$ show a remarkable specificity in DNA interaction, similar to that of cisplatin, being capable of interacting almost exclusively with guanine sequences with G>2 [48 ].

From the compared examination of data of Table 6 and of the effects on experimental tumors it appears that the amount of interstrand cross-linking to DNA and antitumor action are completely separated. In fact, two compounds such as Na[*trans*-$RuCl_4$(DMSO)Im] and Na[*trans*-$RuCl_4$(DMSO)Ox], that exibit a similar pattern of activity on MCa mammary carcinoma *in vivo* (Table 4) and a similar lack of activity against TLX5 lymphoma *in vitro* (Table 3), behave quite differently towards DNA interstrand cross-linking, the former being scarcely effective whereas Na[*trans*-$RuCl_4$(DMSO)Ox] is highly effective, with percentages of interstrand cross-linking similar to those of cisplatin at any D/N ratio tested.

If from one hand the «toxicity» for the metastatic cells seems to fulfill the proposed theory of a more likely activation of ruthenium(III) complexes to cytotoxic products into tumor cells rather than in cells of healthy tissues [20-25], from another point of view it is difficult to explain the significantly lower activity against tumor cells of primary tumors or of lung colonies artificially induced by i.v. injection of tumor cells. A contribution to the antimetastatic action due to an effect at primary site of growth is however observed with Na[*trans*-$RuCl_4$(DMSO)Im], as shown by the reduced metastatic ability of MCa mammary carcinoma in mice transplanted with tumor cells treated *in vivo* with antimetastatic effective doses of **5**. Indeed, such property is evident only at the highest dose used, indicating that this effect can be only in part responsible of the overall activity of Na[*trans*-$RuCl_4$(DMSO)Im] on lung metastasis formation of MCa mammary carcinoma. In fact, the effects at primary site of tumor growth show an unexpected complexity. The reduction of primary tumor growth is only minor and the effects have no long lasting consequence since tumor cells maintain their clonogenic capacity when transplanted into intact syngenic hosts (Sava et al., data on file). Similarly, no evidence of residual effect at this site, as far as metastasizing capacity is concerned, is seen since, after discontinuation of treatments, residual tumors grow and metastasize as no previous treatment had occurred.

Finally, the possibility that the antimetastatic effects of Na[*trans*-$RuCl_4$(DMSO)Im] could be fully ascribed to the differentiating action of dimethylsulphoxide should also be ruled out. In fact **ICR** [27,28], in which the dimethylsulfoxide ligand has been replaced by an imidazole moiety, is as effective as **5** on lung metastases of MCa mammary carcinoma (unpublished results). Indeed, the presence of even small amounts of free DMSO in the solution used for treatments reduces the antitumor activity of Na[*trans*-$RuCl_4$(DMSO)Im]; an action similar to that shown for the structurally related complex [*mer*-$RuCl_3(DMSO)_2NH_3$] [52].

The favourable selective antimetastatic properties described in the previous paragraphs for the ruthenium(III)-dimethylsulfoxide complexes of class **A** in general, and for Na[*trans*-$RuCl_4$(DMSO)Im] in particular, are further stressed by the results obtained testing the therapeutic potential of this compound when combined with surgical excision of primary tumor (Table 7). The high propensity to reduce the growth of lung metastases is evidenced also when treatment occurs after surgical ablation of primary tumor, that is on an advanced stage of growth of the metastatic tumor. In this condition, a treatment schedule can be found that significantly prolongs the life-span of the tumor-bearing animals with an efficacy that is in agreement with the effects of reduction of lung involvement by the metastatic tumor (Table 8).

Again, as shown previously, Na[*trans*-$RuCl_4$(TMSO)Iq] is scarcely active after *in vivo*

treatment also on host's survival time (Table 7).

Table 7. Effects of Na[*trans*-$RuCl_4$(DMSO)Im] and of Na[*trans*-$RuCl_4$(TMSO)Iq] on the survival time of mice bearing MCa mammary carcinoma and undergoing surgical excision of primary tumor.

| Compound | Survival time (days) | | |
|---|---|---|---|
| | mean±S.E. | median | Survivors day 21^ |
| Controls | 26.0±4.2 | 21 | 50% |
| Na[*trans*-$RuCl_4$(DMSO)Im] | 44.9±4.6 | 52 | 100% |
| Na[*trans*-$RuCl_4$(TMSO)Iq] | 22.9±2.9 | 22 | 63% |

Groups of 10 mice, implanted i.m. with $10^6$ cells of MCa mammary carcinoma and undergoing surgical amputation of primary tumor on day 14, were given i.v. 60 mg/Kg/day Na[*trans*-$RuCl_4$(DMSO)Im] or 35 mg/Kg/day Na[*trans*-$RuCl_4$(TMSO)Iq] on days 1,5,9,13.

On the other hand, data on the effects of Na[*trans*-$RuCl_4$(DMSO)Im] on host's survival time are of noteworthy importance in that show the complex capable of a therapeutic activity. They indicate that, unlike many other compounds resulted capable of preventing metastasis formation so far, including Razoxane, DTIC and related triazenes that were active if given before implantation of metastases in the lungs, **5** is effective also when the treatment simulates a rather common clinical situation where drug treatment is applied always after metastatization had already occurred (Table 8). Na[*trans*-$RuCl_4$(DMSO)Im] reduced the growth of established lung metastases and significantly prolonged the life-span of the tumor-bearing animals.

Table 8. Effects of Na[*trans*-$RuCl_4$(DMSO)Im] on lung metastasis formation and on the postsurgical survival time in mice bearing MCa mammary carcinoma and undergoing surgical amputation of primary tumor.

| Treatment group | Lung metastasis weight (mg) | Survival time (days) % ILS |
|---|---|---|
| none | 90.4±14.7 | - |
| 44 mg/Kg/day on days 1-6^ | 19.8±5.7** | 41.7** |
| 44 mg/Kg/day 1hr before surgery | 107.9±19.4 | 2.7 |
| 22 mg/Kg/day on days 1-12 | 41.1±11.0* | 15.7 |

%ILS: percent increase vs controls;
^: given in two separate injections, with 1 hr interval, of 22 mg/Kg/day each;
*: $p<0.05$ and **: $p<0.01$ vs controls.
Groups of CBA mice, inoculated i.m. with $10^6$ MCa mammary carcinoma cells on day 0 and undergoing surgical amputation of primary tumor on day 12, were given Na[*trans*-$RuCl_4$(DMSO)Im] as indicated, starting 24 hr after surgical intervention.

### Conclusions.

The examination of the antitumor potential of ruthenium(III) complexes with dimethylsulfoxide ligands has pointed out their differential effects depending on the tumor and on the site of tumor growth, i.e. primary or metastatic site.

The effects on the survival time correlate with those on lung metastases and indicate the importance of the optimization of parameters such as treatment schedule and daily dosage. Anyhow, the evidence of activity on advanced lung metastases is extremely important in that the model is strictly closed to the most common human situation in which often cancer patients, after successful radical surgery and/or radiotherapy, show the appearance of disseminated metastases that are refractory to conventional cytotoxic treatments because of their nature different from that of the primary tumors from which arose and because the conventional agents actually available have been developed by studies of activity against primary tumors rather than on their metastases. Therefore ruthenium(III)-sulfoxide complexes of class **A**, and Na[*trans*-$RuCl_4$(DMSO)Im] in particular, appear to be a new class of compounds that exibit a selective activity against spontaneous metastases that could be of benefit in the therapy of human neoplasm.

As could be easily expected, class **A** complexes are not able to interact with biological targets as such, but must undergo an activation process, either by reduction or by hydrolisis. All derivatives behave similarly in physiological solution, even though minor differences concerning the rate of hydrolisis of both anionic and neutral ligands have been evidenced, depending on the nature of the nitrogen ligand. Also the redox potential is almost unaffected by the nature of the nitrogen ligand. Parameters such as lipophylicity and steric hindrance of the nitrogen ligand are more likely to be factors of discrimination in the biological behavior of the single derivative, that has been observed both at the level of *in vitro* DNA interactions and of *in vivo* antitumor properties.

It should be also stressed that, despite the apparent similarity between compounds of class **A** and derivatives like **ICR**, the substitution of a nitrogen ligand with a sulfoxide involves rather dramatic changes in foundamental chemical properties of the complex, such as redox potential and rate of hydrolisis of the ligands. Even though, rather surprisingly, the antitumor acivity of the two types of compounds is quite similar in the models examined by us so far, the differences in chemical properties are reflected both in their different capability of interaction with DNA and in the different toxicity and tolerability.

### Acknowledgements

This work was supported by contributions from Italian M.U.R.S.T. (40% and 60% grants), marginally from CNR -special project ACRO, from Boheringer Mannheim Italia and from Fondazione C. e D. Callerio, laboratories for biological research, Trieste.

### References

**1.** *Cisplatin, Current Status and New Developments*: Prestayko,A.W., Crooke,S.T., Carter,S.K. (eds.) London: Academic Press, 1980.

**2.** Loehrer,P.J., Einhorn,L.H., *Ann. Intern. Med.* 1984, **100**, 704-13.

**3.** *Platinum and Other Metal Coordination Compounds in Cancer Chemotherapy*: Nicolini,M. (ed.) Boston (MA): Martinus Nijhoff, 1988.

**4.** *Platinum and Other Metal Coordination Compounds in Cancer Chemotherapy*:

Howell, S.B. (ed.) New York: Plenum Press, 1991.

**5.** Cleare,M.J., Hydes,P.C., in: *Metal Ions in Biological Systems*: Sigel,H. (ed.) New York: Marcel Dekker, 1980; Vol. 11, pp. 1-62.

**6.** Farrell,N., *Transition Metal Complexes as Drugs and Chemotherapeutic Agents*, Dordrecht, The Netherlands: Kluwer Academic Publishers, 1989.

**7.** Haiduc,I., Silvestru,C., *Coord. Chem. Rev.* 1990, **99**, 253-296.

**8.** *Metal Complexes in Cancer Chemotherapy*: Keppler,B.K. (ed.) Weinheim: VCH, 1993.

**9.** Ward,S.G., Taylor,R.C. in: *Metal-Based Anti-Tumor Drugs*: Gielen,M.F. (ed.) London: Freund Publishing House LTD, 1988, pp. 1-54.

**10.** Collery,Ph., Pechery,C. in: *Metal Complexes in Cancer Chemotherapy*: Keppler,B.K. (ed.) Weinheim: VCH, 1993, pp. 249-58.

**11.** Huber,F., Barbieri,R. in: *Metal Complexes in Cancer Chemotherapy*: Keppler,B.K. (ed.) Weinheim: VCH, 1993, pp. 351-68.

**12.** Crowe,A.J. in: *Metal Complexes in Cancer Chemotherapy*: Keppler,B.K. (ed.) Weinheim: VCH, 1993, pp. 369-80.

**13.** Gielen,M., Lelieveld,P., de Vos,D., Willem,R., in: *Metal Complexes in Cancer Chemotherapy*: Keppler,B.K. (ed.) Weinheim: VCH, 1993, pp. 381-90.

**14.** Boyar,E.B., Robinson,S.D., *Coord. Chem. Rev.* 1983, **50**, 109-208.

**15.** Ni Dhubhgaill,O.M., Sadler,P.J., in: *Metal Complexes in Cancer Chemotherapy*: Keppler,B.K. (ed.) Weinheim: VCH, 1993, pp. 221-48.

**16.** Köpf-Maier,P., in: *Progress in Clinical Biochemistry and Medicine*, Berlin: Springer-Verlag, 1989; Vol. 10, pp. 151-184.

**17.** Köpf-Maier,P., in: *Metal Complexes in Cancer Chemotherapy*: Keppler,B.K. (ed.) Weinheim: VCH, 1993, pp. 259-96.

**18.** Heim,M.E., Flechtner,H., Keppler,B.K., in: *Progress in Clinical Biochemistry and Medicine*, Berlin: Springer-Verlag, 1989; Vol. 10, pp. 217-23.

**19.** Keppler,B.K., Friesen,C., Vongerichten, H., Vogel,E., in: *Metal Complexes in Cancer Chemotherapy*: Keppler,B.K. (ed.) Weinheim: VCH, 1993, pp. 297-324.

**20.** Clarke,M.J., in: *Metal Ions in Biological Systems*: Sigel,H. (ed.) New York: Marcel Dekker, 1980; Vol. 11, pp. 231-83.

**21.** Clarke,M.J., Bitler,S., Rennert,D., Buchbinder,M., Kelman,A.D., *J. Inorg. Biochem.* 1980, **12**, 79-87.

**22.** Clarke,M.J., Jansen,B., Marx, K.A., Kruger,R., *Inorg. Chim. Acta* 1986, **124**, 13-28.

**23.** Clarke,M.J., Galang,R.D., Rodriguez,V.M., Kumar,R., Pell,S., Bryan,D.M., in: *Platinum and Other Metal Coordination Compounds in Cancer Chemotherapy*: Nicolini,M. (ed.) Boston (MA): Martinus Nijhoff, 1988, pp. 582-600.

**24.** Clarke,M.J., in: *Progress in Clinical Biochemistry and Medicine*, Berlin: Springer-Verlag, 1989; Vol. 10, pp. 25-39.

**25.** Clarke,M.J. in: *Metal Complexes in Cancer Chemotherapy*: Keppler,B.K. (ed.) Weinheim: VCH, 1993, pp. 129-56.

**26.** Keppler,K.B., Wehe,D., Endres,H., Rupp,W., *Inorg. Chem.* 1987, **26**, 844-46.

**27.** Keppler,B.K., Rupp,W., Juhl,U.M., Endres,H., Niebl,R., Balzer,W., *Inorg. Chem.* 1987, **26**, 4366-70.

**28.** Keppler,B.K., Rupp,W., *J. Cancer Res. Clin. Oncol.* 1986, **111**, 166-68.

**29.** Garzon,F.T., Berger,M.R., Keppler,B.K., Schmähl,D. *Cancer Chemother. Pharmacol.* 1987, **19**, 347-49.

**30.** Keppler,B.K., in: *Progress in Clinical Biochemistry and Medicine*, Berlin: Springer-Verlag, 1989; Vol. 10, pp. 41-69.

**31.** Keppler,B.K., Lipponer,K.-G., Stenzel,B., Kratz,F., in: *Metal Complexes in Cancer Chemotherapy*: Keppler,B.K. (ed.) Weinheim: VCH, 1993, pp. 187-20.

**32.** Gullino,P.M., *Adv. Exp. Biol. Med.* 1976, **75**, 521.

**33.** Palmer,B.D., Wilson,W.R., Pullen,S.M. *J. Med, Chem.* 1990, **33**, 112.

**34.** Srivastava, S.C., Mausner, L.F., Clarke, M.J., in: *Progress in Clinical Biochemistry and Medicine*, Berlin: Springer-Verlag, 1989; Vol. 10, pp. 111-149.

**35.** Kratz,F., in: *Metal Complexes in Cancer Chemotherapy*: Keppler,B.K. (ed.) Weinheim: VCH, 1993, pp. 391-429.

**36.** Reynolds,W.L., *Prog. Inorg. Chem.* 1970, **12**, 1-99.

**37.** Davies,J.A., *Adv. Inorg. Chem. Radiochem.* 1981, **24**, 115-87.

**38.** Sava,G., Zorzet,S., Giraldi,T., Mestroni,G., Zassinovich,G., *Eur. J. Cancer Clin. Oncol.* 1984, **20**, 841-47.

**39.** Alessio,E., Mestroni,G., Nardin,G., Attia,W.M., Calligaris,M., Sava,G., Zorzet,S. *Inorg. Chem.* 1988, **27**, 4099-4106.

**40.** Alessio, E., Attia,W.M., Calligaris,M., Cauci,S., Dolzani,L., Mestroni,G., Monti-Bragadin,C., Nardin,G., Quadrifoglio,F., Sava,G., Tamaro,M., Zorzet,S., in: *Platinum and Other Metal Coordination Compounds in Cancer Chemotherapy*: Nicolini,M. (ed.) Boston (MA): Martinus Nijhoff, 1988, pp. 617-33.

**41.** Alessio,E., Xu,Y., Cauci,S., Mestroni,G., Quadrifoglio,F., Viglino,P., Marzilli,L.G., *J. Am. Chem. Soc.* 1989, **111**, 7068-71.

**42.** Mestroni,G., Alessio,E., Calligaris,M., Attia,W.M., Quadrifoglio,F., Cauci,S., Sava,G., Zorzet,S., Pacor,S., Monti-Bragadin,C., Tamaro,M., Dolzani,L., in: *Progress in Clinical Biochemistry and Medicine*, Berlin: Springer-Verlag, 1989; Vol. 10, pp. 71-87.

**43.** Sava,G., Pacor,S., Zorzet,S., Alessio,E., Mestroni,G., *Pharmacol. Res.* 1989, **21**, 617-28.

**44.** Sava,G., Pacor,S., Bregant,F., Ceschia,V., Luxich,E., Alessio,E., Mestroni,G., *Pharmacol. (Life Sci. Adv.)* 1990, **9**, 79-84.

**45.** Loseto,F., Alessio,E., Mestroni, Lacidogna,G., Nassi, A., Giordano,D., Coluccia,M., *Anticancer Res.*, 1991, **11**, 1549-54.

**46.** Esposito, G., Cauci,S., Fogolari, F., Alessio, E., Scocchi, M., Quadrifoglio, F., Viglino,P. *Biochemistry*, 1992, **31**, 7094-7103.

**47.** Colucci,M., Coluccia,M., Montemurro,P., Conese,M., Nassi,A., Loseto,F., Alessio,E., Mestroni,G., Semeraro,N., *Int. J. Oncol.*, 1993, *2*, 527-29.

**48.** Coluccia,M., Sava,G., Loseto,F., Nassi,A., Boccarelli,A., Giordano,D., Alessio,E., Mestroni,G., *Eur. J. Cancer*, in press.

**49.** Alessio,E., Milani,B., Mestroni,G., Calligaris,M., Faleschini,P., Attia,W.M., *Inorg. Chim. Acta* 1990, **177**, 255-65.

**50.** Costa,G., Balducci,G., Alessio,E., Tavagnacco,C., Mestroni,G., *J. Electroanal. Chem.* 1990, **296**, 57-76.

**51.** Alessio,E., Balducci,G., Calligaris,M., Costa,G., Attia,W.M., Mestroni,G., *Inorg. Chem.* 1991, **30**, 609-18.
**52.** Pacor,S., Sava,G., Ceschia,V., Bregant,F., Mestroni,G., Alessio,E., *Chem.-Biol. Interact.*, 1991, **78**, 223-34.
**53.** Sava,G., Pacor, S., Mestroni,G., Alessio,E., *Anti-Cancer Drugs*, 1992, **3**, 25-31.
**54.** Sava,G., Pacor,S., Mestroni,G., Alessio,E., *Clin. Exp. Metastasis*, 1992, **10**, 273-80.
**55.** Alessio, E., Balducci, G., Lutman, A., Mestroni, G., Calligaris, M., Attia, W.M., *Inorg. Chim. Acta* 1993, **203**, 205-217.
**56.** Mestroni, G., Alessio, E., Sava, G., Pacor, S., Coluccia, M., in: *Metal Complexes in Cancer Chemotherapy*: Keppler,B.K. (ed.) Weinheim: VCH, 1993, pp. 157-86.
**57.** Howard, R. A., Sherwood, E., Erck, A., Kimball, A. P., Bear, J. L., *J. Med. Chem.* 1977, **20**, 943-46.
**58.** Taqui Khan, M. M., Ramachandraiah, G., Prakash Rao, A., *Inorg. Chem.* 1986, **25**, 665-70.
**59.** McMillan, R. S., Mercer, A., James, B. R., Trotter, J. *J. Chem. Soc. DaltonTrans.*, 1975, 1006.
**60.** Cauci,S., *Ph.D. Dissertation Thesis*, University of Udine, Udine, Italy.
**61.** Talmadge, J.E., *Cancer Metastasis Rev.*, 1983, **2**, 25-40.
**62.** Heppner, G.H., Miller, B.E., *Cancer Metastasis Rev.*, 1983, **2**, 5-24.
**63.** Morgan, A.R., Pulleybank, D.E., *Biochem. Biophys. Res. Commun.* 1974, **61**, 396-403.
**64.** Brent, T.O., *Cancer Res.* 1984, **44**, 1887-92.

**Received: August 2, 1993 - Accepted: August 14, 1993**

